# Loss of E-cadherin activates EGFR-MEK/ERK signaling, which promotes invasion via the ZEB1/MMP2 axis in non-small cell lung cancer

**DOI:** 10.18632/oncotarget.1463

**Published:** 2013-11-29

**Authors:** Gab-Yong Bae, So-Jung Choi, Ji-Seon Lee, Jisuk Jo, Jinseon Lee, Jhingook Kim, Hyuk-Jin Cha

**Affiliations:** ^1^ Department of Life Science, Sogang University, Seoul, Republic of Korea.; ^2^ Samsung Biomedical Research Institute, Samsung Medical Center, Sungkyunkwan University, School of Medicine, Seoul, Republic of Korea.; ^3^ Department of Thoracic Surgery, Samsung Medical Center, Sungkyunkwan University, School of Medicine, Seoul, Republic of Korea.; ^4^ Samsung Genome Institute, Research Institute for Future Medicine, Samsung Medical Center.

**Keywords:** E-Cadherin, EGFR-MEK/ERK signaling, ZEB1, MMP2, Invasion

## Abstract

Loss of E-cadherin, a hallmark of epithelial-mesenchymal transition (EMT), can significantly affect metastatic dissemination. However, the molecular mechanism of EMT-associated metastatic dissemination by loss of E-cadherin still remains unclear in non-small cell lung cancers (NSCLCs). In the present study, we show that the knockdown of E-cadherin was sufficient to convert A549 NSCLC cells into mesenchymal type with the concurrent up-regulation of typical EMT inducers such as ZEB1 and TWIST1. Interestingly, the EMT-induced cells by E-cadherin depletion facilitate invasion in a matrix metalloproteinase-2 (MMP2)-dependent manner with aberrant activation of EGFR signaling. We demonstrated that the elevated invasiveness was a result of the activated EGFR-MEK/ERK signaling, which in turn leads to ZEB1 dependent MMP2 induction. These results suggest that the EGFR-MEK/ERK/ZEB1/MMP2 axis is responsible for promoted invasion in EMT-induced NSCLCs. Consistently, ERK activation and loss of E-cadherin were both observed in the disseminating cancer cells at the invasive tumor fronts in NSCLC cancer tissues. Thereby, these data suggest that the EGFR-MEK/ERK signaling would be a promising molecular target to control aberrant MMP2 expression and consequent invasion in the EMT-induced NSCLCs

## INTRODUCTION

EMT is a highly conserved developmental process, in which a polarized epithelial cell acquires the properties of a mesenchymal cell during embryonic development [1]. However, besides role during development, EMT is well-characterized in malignant tumor progression, metastasis [2] and even acquisition of cancer stemness [3]. E-cadherin, a member of the cadherin superfamily, is involved in maintaining cell polarity and organizing the epithelium by strengthening intercellular adhesion; thus E-cadherin can act as a suppressor of invasion. Therefore, the loss of E-cadherin expression, a prototypical marker of EMT [2], is frequently found in various metastatic human epithelial cancers [4].

Of note, the promotion of metastasis by the loss of E-cadherin has been shown to result from the activation of intracellular signaling and subsequent up-regulation of transcription factors [5, 6]. Particularly, the aberrant activation of epidermal growth factor receptor (EGFR) signaling contributes to NSCLC progression and is highly correlated with poor prognosis [7]. In line with this, EGFR ligands such as epidermal growth factor (EGF) and transforming growth factor-α (TGFα) are frequently overexpressed in NSCLC patients [8]. Other than gene amplification [9] or mutation of EGFR itself [7], activation of EGFR signaling is closely associated with the status of E-cadherin. However, the positive or negative role of E-cadherin in EGFR dependent signaling remains controversial despite a number of independent studies [5, 10-13].

In this study, we found that the EGFR-MEK/ERK signaling is aberrantly activated by simple depletion of E-cadherin, and is also closely associated to invasion in MMP2 dependent manner. A closer examination revealed that the ZEB1, of which expression is regulated by EGFR-MEK/ERK signaling, is responsible for the invasive property through upregulation of MMP2. Consistently, the positive correlation between loss of E-cadherin and ERK activation was also observed in the disseminating cancer cells of NSCLC patients, demonstrating that the EGFR-MEK/ERK axis would be a promising target for attenuating deviant MMP2 expression and the subsequent invasive trait in EMT-induced NSCLCs.

## RESULTS

### Knockdown of E-cadherin is sufficient to induce EMT

**Figure 1:**
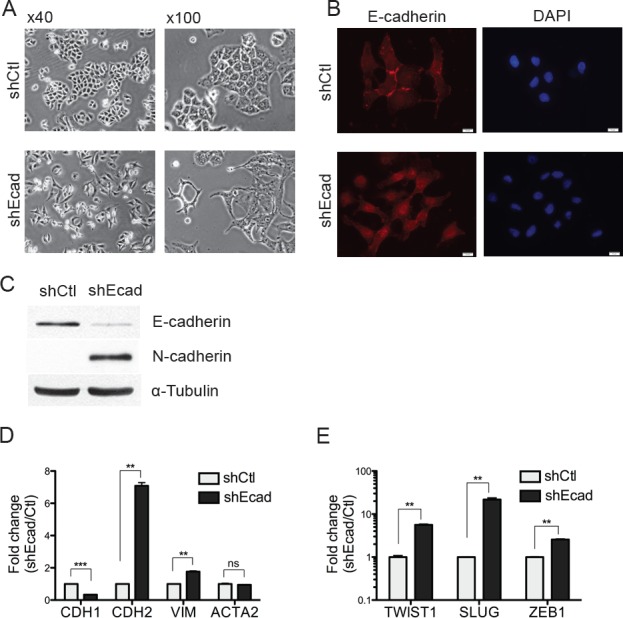
Knockdown of E-cadheirn induces the EMT in A549 cells

To investigate the effect of E-cadherin on the EMT process in A549 NSCLC cells, the E-cadherin knockdown cell line (E-cad KD: shEcad) was generated by the stable expression of E-cadherin shRNA. The E-cad KD A549 cells showed dramatic morphological changes compared to the shRNA control A549 cells (shCtl). The mesenchymal features such as loss of cell polarity, spindle-like cell shape and loss of cell-to-cell adhesion were distinct in shEcad, whereas the epithelial characteristics such as close cell-to-cell adhesion and cobblestone-like cell shape were still observed in shCtl (Fig. 1A). Additionally, the E-cadherin signal clearly observed at the boundaries of shCtl was abolished in shEcad, indicating that altered cell-to-cell adhesion in shEcad is associated with the loss of E-cadherin (Fig. 1B). In the characteristic EMT process, loss of E-cadherin is accompanied with gain of N-cadherin, which is known as the ‘E- to N-cadherin switching’ [4] (Fig. 1C). In addition to morphological changes, mesenchymal genes such as *CDH2* (encoding N-cadherin) and *VIMENTIN* (encoding Vimentin) but not *ACTA2* (encoding α-smooth muscle actin; SMA) were clearly upregulated (Fig. 1D). Of note, a simple E-cadherin knockdown was able to promote the expression of several transcription factors such as *TWIST1*, *SLUG,* and *ZEB1*, which serve as typical EMT inducers [14] (Fig. 1E). This result is in agreement with an earlier study in breast cancer cells showing that the loss of E-cadherin is sufficient to alter the wide range of transcriptional changes, including changes in *TWIST1* and *ZEB1* [6], implying that both *TWIST1* and *ZEB1* could be the common EMT-inducing regulators in E-cadherin knockdown cancer cells.

A. Representative images of cells were taken at 40x and 100x magnification respectively. B. Cells were stained with E-cadherin antibody (red) and counterstained with DAPI (blue). Scales, 10 μm. C. Total cell lysates were subjected to western blot. α-tubulin was used as a loading control. D and E. The transcript levels of mesenchyme-related genes (D) and EMT-inducing genes (E) were measured using Real-time PCR. *ACTB* (Beta Actin) was used as an internal control for relative quantification of gene expression. *Columns*, means ± SE. *P < 0.05; **P < 0.01; ***P < 0.001.

### Loss of E-cadherin promotes invasion, which is dependent on increased MMP2 expression

**Figure 2:**
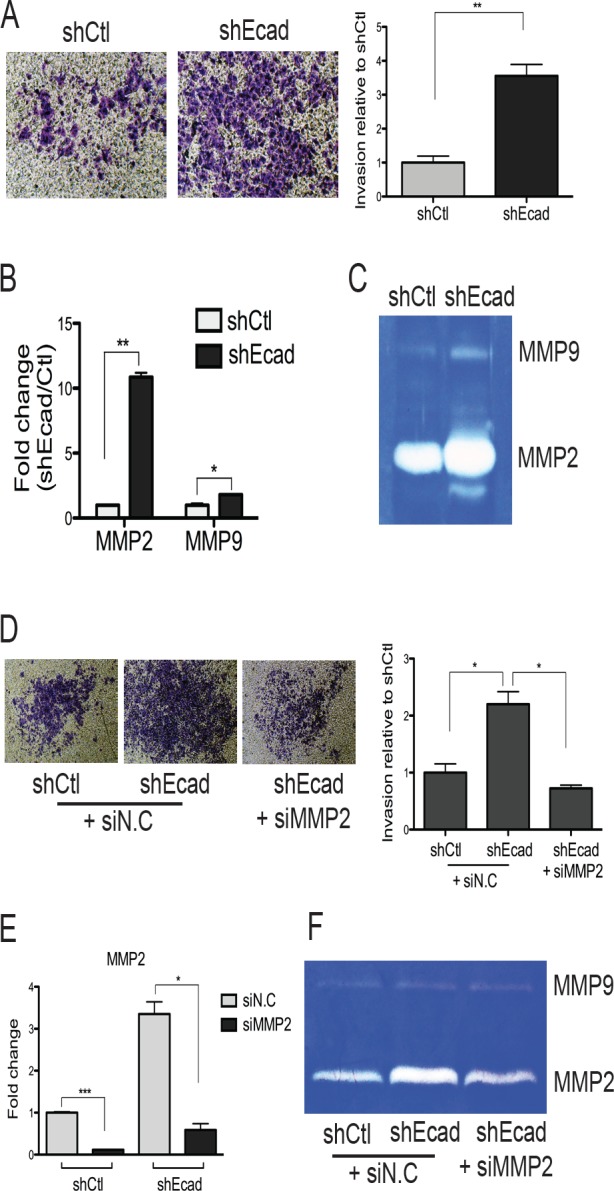
Enhanced invasiveness is attributed to elevated MMP2 expression in shEcad

E-cadherin knockdown cells showed typical mesenchymal phenotypes [3, 15, 16] such as strong cell-to-matrix adhesion (data not shown), slight growth retardation (Fig. S1A) and increased ability of tumor sphere formation (Fig. S1B). Notably, a loss of proliferative activity is also one of the features associated with EMT during tumor cell dissemination [16], and the tumor sphere formation is a feature of cancer stem-like cells [17], which could be generated as a result of EMT [18]. Most significantly, the invasive property of shEcad cells was markedly increased when compared to the control cells (Fig. 2A). The invasion assay system employed in our study aimed at evaluating the level of invading cells across the matrigel-embedded membrane, which mimics the extracellular matrix (ECM). Thereby, the increased invasion in shEcad may result from the elevated proteolytic action on the ECM component. For this reason, we examined the level of matrix metalloproteinases (MMPs), which are critical for tumor metastasis by regulating invasion, angiogenesis, apoptosis, inflammation, cell growth, and the metastatic niche [19]. Initially, we focused on MMP2 and MMP9, because high expression of *MMP2* or *9* is commonly observed in various cancers including lung cancer, and their expression is positively correlated with tumor malignancy and poor diagnosis of cancer patients [20]. As predicted, the mRNA level of both *MMP2* and *MMP9* increased in shEcad, with *MMP2* being more significant (Fig. 2B). Consistent with the expression pattern of *MMP2* and *9*, the enzymatic activity of MMP2, determined by zymography, showed a robust increase in shEcad whereas that of MMP9 increased only slightly (Fig. 2C). To demonstrate that the invasive phenotype in shEcad (Fig. 2A) is a result of MMP2 upregulation (Fig. 2B), the level of invasion was compared after *MMP2* knockdown. A significant reduction in the invasive phenotype of shEcad was observed by *MMP2* knockdown (Figs. 2D and E), which was concurrent with a reduction in the MMP2 activity but not the MMP9 activity (Fig. 2F).

A. Cells were allowed to invade through Matrigel-coated trans-well membranes. The migrated cells were fixed and stained with crystal violet. The representative images are shown (left panels). Invasiveness was quantified by measuring the stained areas and fold changes are presented as a bar graph (right panel). B and C. The transcript levels (B) and the enzymatic activities (C) of MMP2 and MMP9 were determined with Real-time PCR and zymography respectively. D-F. Invasion assay was performed two days after knockdown of MMP2. Cells were transfected with negative control siRNA (siN.C) and *MMP2* siRNA (siMMP2). Representative images of invaded cells are shown (left panels), and fold changes of invaded area at each condition are represented as a bar graph (D). *Columns*, means ± SE. *P < 0.05. The knockdown level of *MMP2* by siMMP2 was determined by Real-time PCR analysis (E) and zymography (F). *Columns*, means ± SE. *P < 0.05; **P < 0.01; ***P < 0.001.

### EGFR signaling is activated in E-cadherin knockdown A549 cells

Although singly migrating cancer cells have high motility, thereby being beneficial on metastatic dissemination, many invasive tumors exhibit collective invasion, in which multicellular aggregates infiltrate into the peritumoral stroma, while maintaining the cell-to-cell adhesion [21-23]. This indicates that cell-cell adhesion is not a sole barrier against invasion. Recently, the role of E-cadherin as a modulator of intracellular signaling was emphasized in cancer metastasis [6], implying that E-cadherin inhibits invasion not only by strengthening cell-cell adhesion but also by suppressing pro-invasive intracellular signaling. Thus we hypothesized that the loss of E-cadherin might result in changes in intracellular signaling, which can lead to the altered gene expression (Figs. 1D and E) and ultimately trigger invasion (Fig. 2A).

**Figure 3:**
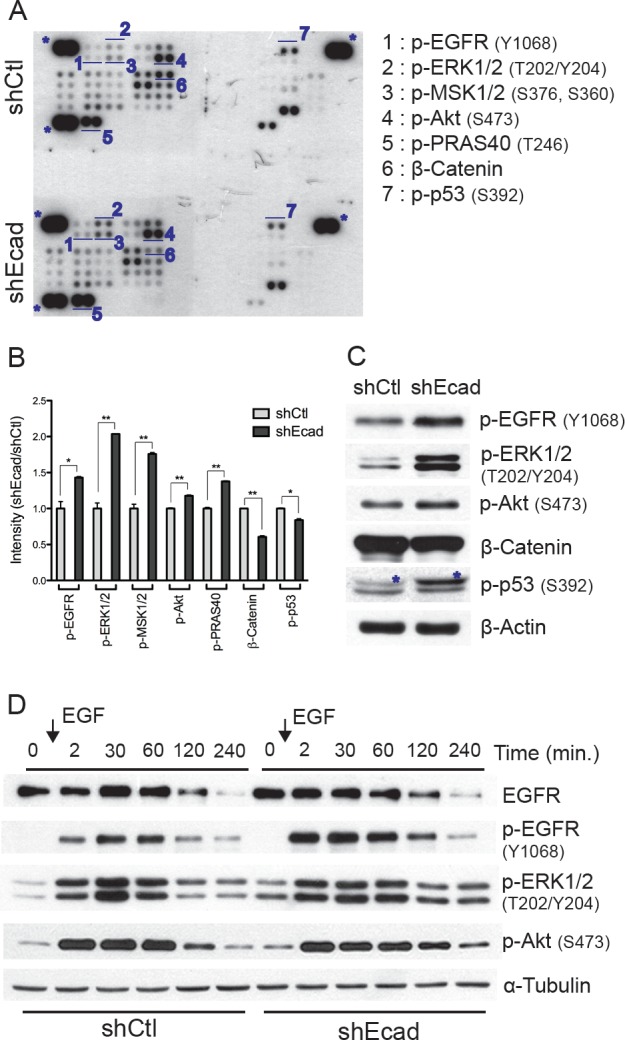
Loss of E-cadherin leads to activation of EGFR signaling

As protein kinases are critical regulators of intracellular signaling pathways [24], the deregulation of these kinases contributes to promoting tumorigenesis [25]. The ‘phospho-proteome profiler’ assay allowed us to survey the levels of phosphorylation of 43 kinases that are crucial for regulating the tumorigenic properties. Among them, we observed that EGFR dependent downstream signaling was distinctly upregulated in the shEcad (Fig. 3). Phosphorylation of EGFR itself (Y1068) and EGFR downstream kinases such as Akt (S473) and ERK1/2 (T202/Y204) were increased in shEcad. Consistent with this result, phosphorylation levels of Mitochondrial lysine-tRNA synthetase 1/2 (MSK1/2) (S376/S360) and Protein-rich Akt1 substrate of 40 kDa (PRAS40) (T246), which are substrates of ERK1/2 [26] and Akt [27] respectively, were elevated in shEcad (Figs. 3A and B). The results of phospho-proteome profiler assay were validated by immunoblotting (Fig. 3C). These data indicate that EGFR and its downstream kinases such as ERK and Akt are activated following loss of E-cadherin.

A. Cell lysates were incubated with membranes spotted with phospho-specific antibodies against 43 kinases. Each antibody is spotted in duplicate. Spots representing the key signaling molecule were labeled with numbers (1-7) and their corresponding antibodies were shown. Asterisk indicates reference spots. B. The intensities of spots were measured using Image J and fold changes are presented as a bar graph. C. Altered signals were verified by immunoblotting analysis with corresponding antibodies. Asterisks indicate nonspecific bands. D. Serum-starved cells were stimulated with EGF (30 ng/ml) and harvested at indicative time, and then were subjected to immunoblotting analysis. Phosphoryated levels of EGFR, ERK1/2 and Akt were determined. α-tubulin was used as a loading control. *Columns*, means ± SE. *P < 0.05; **P < 0.01.

As the physical interaction of E-cadherin with EGFR negatively regulates ligand-dependent activation [13], the increased phosphorylation of EGFR and its downstream kinases may result from higher ligand responsiveness in the absence of E-cadherin. To prove this hypothesis, EGF responsiveness was compared between shCtl and shEcad after serum starvation, during which growth factor-dependent signaling is reduced to the basal level. In both shCtl and shEcad, the phosphorylation levels of EGFR, ERK and Akt promptly increased upon EGF stimulation and then gradually decreased, while the total EGFR expression decreased after EGF stimulation, which might be the consequence of EGF-stimulated EGFR endocytosis [28]. Interestingly, shEcad exhibited a more rapid and persistent response to EGF stimulation than shCtl. In shEcad cells, EGFR phosphorylation was more rapidly saturated in response to EGF, and the elevated phosphorylation of EGFR and its downstream kinases (ERK and Akt) was persistently maintained as compared to shCtl cells (Fig. 3D). Considering that a single transient stimulation with EGF resulted in a significant upregulation of EGFR-dependent signaling, the prominent activation of EGFR and its downstream signaling in shEcad (Figs. 3A-C) would be the result of increased sensitivity of EGFR to EGF stimulation. These results suggest that the loss of E-cadherin promotes EGFR-dependent signaling toward MEK/ERK and PI3K/Akt pathways through increased ligand responsiveness of EGFR.

### EGFR-MEK/ERK signaling regulates the expression of *TWIST1*, *ZEB1* and *MMP2*

**Figure 4:**
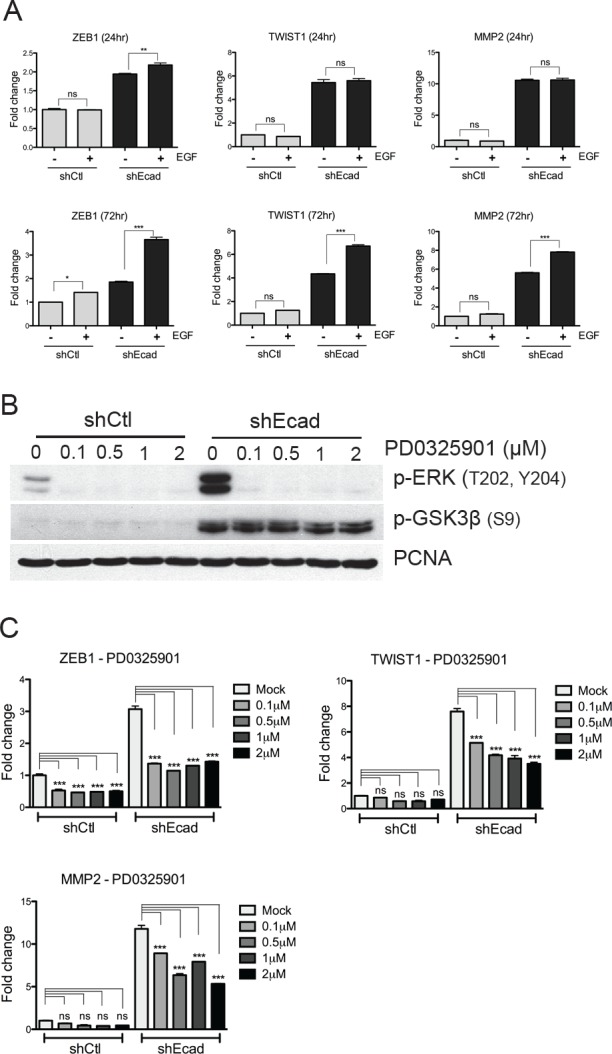
EGFR-MEK/ERK signaling up-regulates the expression of EMT-associated genes

A pronounced difference between shEcad and shCtl cells is the increased expression of *ZEB1*, *TWIST1*, and *MMP2* (Figs. 1E and 2B), all of which are involved in metastasis [3, 29] and belong to ‘EMT-associated genes’ [30]. Subsequently, we examined whether the expression of these genes is associated with higher EGF responsiveness of shEcad. For this purpose, the mRNA levels of *ZEB1*, *TWIST1*, and *MMP2* were determined following transient activation of EGFR-dependent signaling. Interestingly, shEcad exhibited higher responsiveness toward EGF stimulation, both at 24 hr (*ZEB1*) and 72 hr (*TWIST1, ZEB1 and MMP2*), compared to the shCtl cells (Fig. 4A).

A. Cells were treated with EGF (30 ng/ml). The EGF-containing media were freshly changed every 24 hours. Cell lysates were extracted to analyze mRNA levels of *ZEB1*, *TWIST1* and *MMP2* at 24 hours (top panels) and 72 hours (bottom panels) with Real time PCR. B and C. Cells were treated with indicated concentration of PD0325901, a MEK inhibitor for 4 hours (B) or 24 hours (C) and the cell lysates were harvested for protein analysis (B) or RNA analysis (C). Effect of PD0325901 in MEK/ERK signaling was determined by immunoblotting against phospho-ERK, a substrate of MEK (B). PCNA was used as a loading control. Note that MEK inhibition significantly reduced the expression of *ZEB1*, *TWIST1* and *MMP2* in shEcad (C). *Columns*, means ± SE. **P < 0.01; ***P < 0.001; ns, non-significant.

The EGFR-dependent gene expression could primarily be mediated by the MEK/ERK or the PI3K/Akt signaling, or cooperatively by the two pathways. To determine the individual contribution of the two pathways in EGFR signaling-dependent expression of the EMT-associated genes, we took advantage of PD0325901 (PD) or MK2206 (MK), which selectively inhibit MEK1/2 [31] and Akt [32] respectively. In both shCtl and shEcad cells, active phosphorylation of ERK1/2 but not GSK3β was noticeably decreased even at a low concentration of PD (0.1 μM) (Fig. 4B). Concurrently, *ZEB1*, *TWIST1* and *MMP2* were considerably downregulated by PD treatment in a dose-dependent manner in shEcad, whereas only *ZEB1* expression was reduced in shCtl (Fig. 4C). In contrast to the results with PD treatment, Akt inhibition by MK treatment, determined by the level of active phosphorylation of Akt and inhibitory phosphorylation of GSK3β (Fig. S2A), did not significantly affect the expression of either *ZEB1* or *MMP2*, and had only a marginal effect on *TWIST1* expression (Fig. S2B). These data indicate that higher and/or more sustained activation of EGFR in shEcad cells follows the MEK/ERK signaling axis rather than PI3K/Akt, which in turn, elevates the levels of *ZEB1* and *MMP2*, while both MEK/ERK and PI3K/Akt signaling cooperatively regulate *TWIST1* expression.

### MEK/ERK signaling is required for the invasive phenotype of shEcad

**Figure 5:**
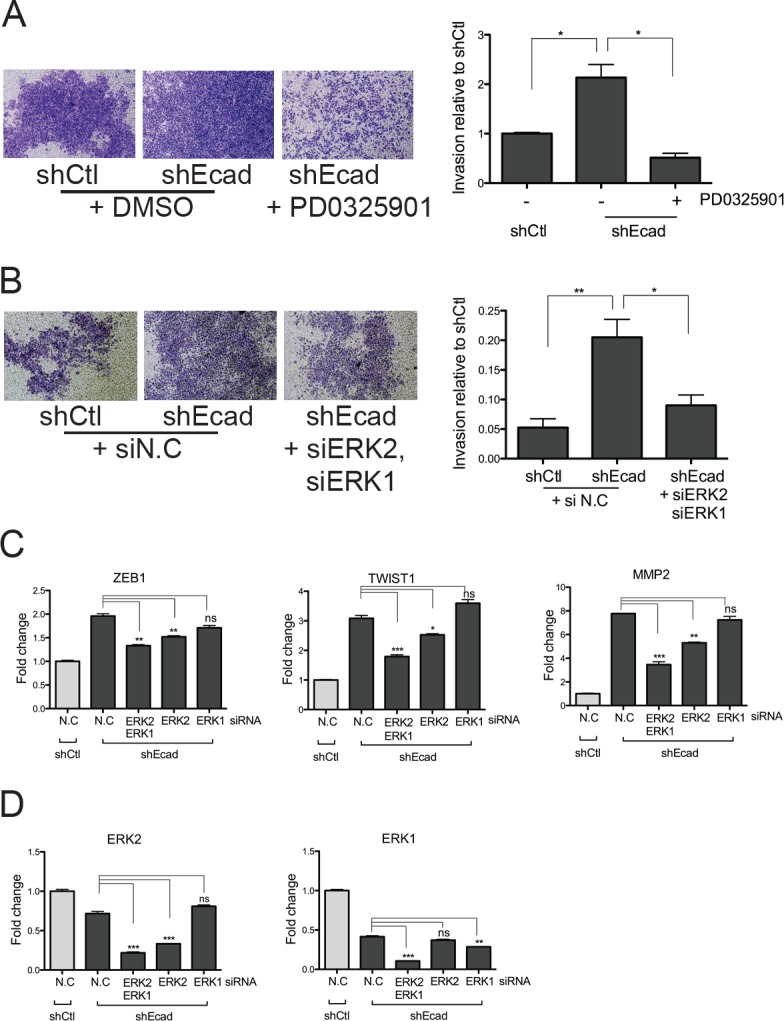
Inhibition of MEK/ERK signaling reduces the EMT phenotypes of shEcad

On the basis of MEK/ERK signaling-dependent *MMP2* induction (Fig. 4C), which was closely associated with the elevated invasive behavior of shEcad cells (Fig. 2), it is readily surmised that the invasiveness of shEcad may result from an activated MEK/ERK axis due to higher and/or more sustained EGFR activation. To examine this hypothesis, the invasive activity of shEcad cells was determined following PD treatment. As predicted, the enhanced invasion of shEcad cells (Fig. 2A) was noticeably abolished by a single treatment of PD (Fig. 5A). To rule out non-specific effects of PD, other than inhibition of MEK activity toward ERK, ERK1 and 2 were depleted with siRNAs to monitor the invasive character in shEcad. Consistent with the results observed with PD treatment, simultaneous depletion of ERK1 and 2 clearly attenuated the enhanced invasive property of shEcad (Fig. 5B). As the enhanced invasive property of shEcad results from the induction of MMP2 (Fig. 2), we predicted that the reduced invasion of shEcad after PD treatment (Fig. 5A) or ERK1 and 2 depletion (Fig. 5B) was accompanied with the suppression of MMP2, the expression of which is in turn dependent on the MEK/ERK axis (Fig. 4C).

A and B. Cells were treated with PD0325901 (0.5 μM) (A) or siRNA (B) for 48 hours and allowed to invade through Matrigel-coated trans-well membranes. Negative control siRNA (siN.C), ERK1 siRNA (siERK1), and ERK2 siRNA (siERK2) were used. Representative images (left panels) and quantification of the invaded area (right panels) are represented. C and D. Cells were treated with negative control (N.C) siRNA, ERK2 siRNA, and/or ERK1 siRNA. After 24 hour incubation, cells were changed to fresh media with 0.5% FBS for 48 hours. Total RNA was extracted from cells, and mRNA levels of *ZEB1*, *TWIST1*, and *MMP2* were determined with Real-time PCR (C). Knockdown efficiency of ERK1 or ERK2 was determined by Real-time PCR analysis (D). *Columns*, means ± SE. *P < 0.05; **P < 0.01; ***P < 0.001; ns, non-significant.

Between ERK1 and ERK2, ERK2 rather than ERK1 showed a more prominent effect on *MMP2* induction (Fig. 5C), while both ERK1 and ERK2 were efficiently knocked down by their corresponding siRNAs (Fig. 5D). However, it is noteworthy that the simultaneous depletion of ERK1 and 2 more effectively reduced *MMP2* levels than the depletion of ERK2 alone (Fig. 5C). Similarly, the expression of both *TWIST1* and *ZEB1* was also dependent on ERK1/2, with ERK2 being dominant over ERK1 (Fig. 5C). The higher dependency of *TWIST1, ZEB1*, and *MMP2* on ERK2 rather than ERK1 concurs with previous studies demonstrating that ERK1 fails to compensate for the critical roles of ERK2 during embryonic development [33, 34]. Our data suggest that the elevated MEK/ERK signaling following hyper-reactivity to EGF in the EMT cells promotes invasion by inducing the EMT-associated genes, particularly *MMP2*. Additionally, it is noticeable that ERK2 functions as an important signal transducer in regulating MMP2-mediated invasion, while ERK1 may assist in fine-tuning the ERK2 activity, as was previously suggested [35]. Our findings, which demonstrate that ERK induces EMT or cancer invasion, suggest a pro-tumorigenic function of ERK and are supported by other studies [36, 37].

### ZEB1 is responsible for MMP2 induction

**Figure 6:**
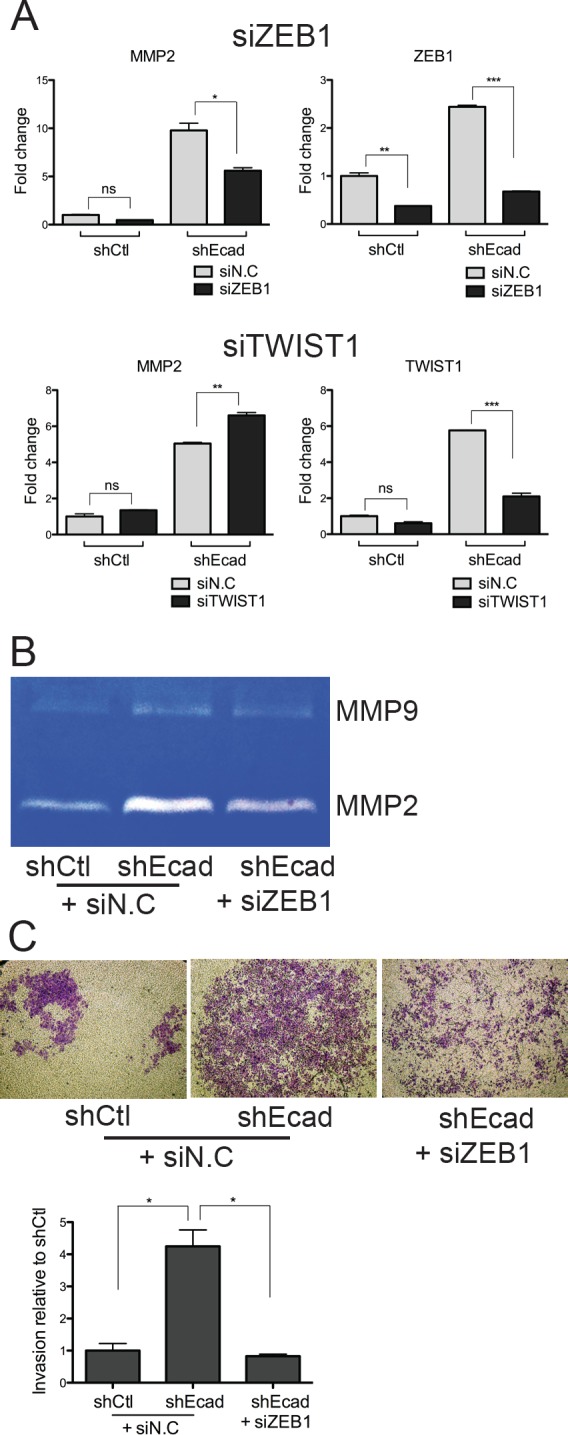
Knockdown of *ZEB1* reduces MMP2 expression and invasive properties

We next examine the link of ZEB1 or TWIST1 with MMP2, of which expression is dependent on ERK activity (Fig. 5). For this purpose, we transiently knocked down the expression of *ZEB1* or *TWIST1* with siRNA and then monitored *MMP2* expression, which was responsible for the invasive property of EMT cells (Fig. 2A). Depletion of *ZEB1* transcripts, but not *TWIST1*, resulted in a reduction of the *MMP2* transcript (Fig. 6A). Consistently, the increased MMP2 enzymatic activity in shEcad was clearly diminished following *ZEB1* depletion, whereas MMP9 was less affected (Fig. 6B). Moreover, the invasiveness of shEcad was severely impaired by *ZEB1* knockdown (Fig. 6C).

A. Cells were treated with negative control siRNA (siN.C), *ZEB1* siRNA (siZEB1), and *TWIST1* siRNA (siTWIST1) for 48 hours and *MMP2* mRNA level was analyzed by Real-time PCR. B and C. Cells were treated with negative control siRNA (siN.C), and *ZEB1* siRNA (siZEB1) for 48 hours. The conditioned medium of cells was subjected to zymography (B), and cells were subjected to invasion assay (C). Representative images (upper panels) and quantification of the invaded area (lower panel) are represented. *Columns*, means ± SE. *P < 0.05; **P < 0.01; ***P < 0.001; ns, non-significant.

Taken together, the elevated *ZEB1* expression following EMT may serve as an important mediator that could converge highly activated EGFR-MEK/ERK signaling on *MMP2* induction, facilitating the invasion of the EMT-induced cancer cells.

#### Local activation of ERK in the EMT-associated disseminating tumor cells

**Figure 7:**
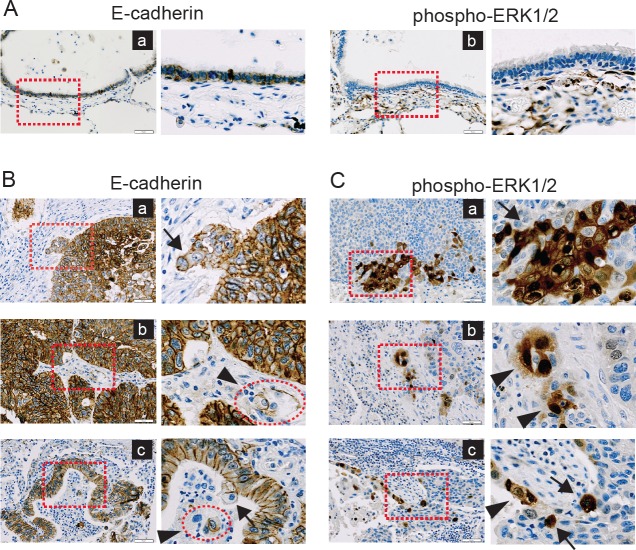
Disseminating tumor cells at the marginal regions of tumors exhibit loss of E-cadherin and ERK activation

As the aberrant ERK activation is considered as an important signature in the EMT-associated invasion process, as well as the expression of *ZEB1*, *TWIST1* and *MMP2* in *in vitro* model, we assumed that the status of E-cadherin and ERK is an applicable index to represent the EMT-associated phenotypes in NSCLCs. To validate this idea, we analyzed the expression pattern of E-cadherin and phospho-ERK1/2 in 34 tissue specimens of NSCLC patients to assess the relationship between loss of E-cadherin and the extent of activated ERK1/2. In normal lung tissues, E-cadherin expression was distinct, whereas phospho-ERK1/2 staining was not observed in the normal epithelial cells (Fig. 7A). In cancer tissues, the staining patterns of E-cadherin and phospho-ERK1/2 appeared to be more complicated. Like normal tissues, tumor cells exhibited well-organized epithelial phenotypes, showing close cell-to-cell adhesion and abundant expression of E-cadherin (Fig. 7B). As similar as shown in a previous report [16], however, a close examination revealed that loss of E-cadherin expression frequently observed at the locally advancing marginal regions of tumors, wherein “detachment of small cell clusters” [16] was observed (Fig. 7B, red square boxes). Of note, E-cadherin expression was partially or completely lost in ‘detaching tumor cells’ (Fig. 7B, arrows) and ‘isolated tumor cells’ (Fig. 7B, arrowheads), which is consistent with the case of ‘disseminating tumor cells’ at the invasive tumor fronts [16]. When the tumor marginal regions (22 cases) were more closely examined, loss of E-cadherin was observed in all of the disseminating tumor cells (e.g. detaching tumor cells and isolated tumor cells). Of note, the disseminating tumor cells appeared to lose cell polarity and orientation, indicating EMT phenotypes. In agreement with a previous study [16], our observation suggested that the loss of E-cadherin at the invasive tumor fronts, which is accompanied by acquisition of mesenchyme-like phenotypes, might be necessary for the early stage of metastasis, including dissociation, invasion and migration.

Immunohistochemical analyses of NSCLC tissues were performed with E-cadherin (left panels) or phospho-ERK (T202, Y204) (right panels) antibody. Representative images were taken at 200x magnification (left side of each panel) and the regions marked with red square boxes were represented with higher magnification (right side of each panel). Scale bar, 50 μm. Nucleus was counterstained by hematoxylin. A. Normal tissues stained for E-cadherin or phospho-ERK1/2 (T202, Y204). Epithelial cells were positive for E-cadherin staining (left panels) and negative for phospho-ERK1/2 staining (right panels). B. Tumor tissues stained for E-cadherin. E-cadherin expression was clearly detected in inner (or central) regions of tumors, but not in disseminating regions of tumors (red square boxes) (a-c). Distinct loss of E-cadherin was observed in detaching tumor cells (arrows) and isolated tumor cells (arrowheads). The isolated tumor cells formed a small cell cluster (red circular boxes) C. Tumor tissues stained for phospho-ERK1/2 (T202, Y204). Staining for phospho-ERK1/2 was negative in inner (or central) regions of tumors, but positive in disseminating regions of tumors (red square boxes) (a-c). In the disseminating regions, distinct ERK activation was detected in the detaching tumor cells (arrows) and isolated tumor cells (arrowheads).

In contrast to E-cadherin, the staining of active ERK1/2 was even more locally restricted (Fig. 7C). Phospho-ERK1/2 was absent in the central regions of tumors, whereas the distinguishable staining for phospho-ERK1/2 was restrained at the disseminating regions of tumors (Fig. 7C, red square boxes). Importantly, clear signal for phospho-ERK1/2 was observed in the detaching tumor cells (Fig 7C, arrows) and the isolated tumor cells (Fig 7C, arrowheads), indicating that ERK is activated in the disseminating tumor cells, in which mesenchymal phenotypes such as loss of E-cadherin expression and loss of cell polarity are acquired (Fig. 7B). Thus, these staining patterns showed the positive correlation between loss of E-cadherin and ERK activation, which was consistent with the *in vitro* cell model. Furthermore, these results indicate that the disseminating tumor cells at the invasive marginal regions of tumors may undergo EMT, when loss of E-cadherin induces ERK activation through deregulated EGFR activation, eventually promoting invasion.

## DISCUSSION

The role of E-cadherin in regulating oncogenic signaling that governs the EMT process has received tremendous attention, demonstrating that the loss of E-cadherin is sufficient to induce EMT, ultimately leading to metastasis [2]. NSCLC patients with metastases frequently show deregulation of EGFR, including high expression or gain of function mutations, which are correlated with poor prognosis [8, 9]. Thus, the mutational status or expression levels of E-cadherin and EGFR have to be considered for the efficient treatment of NSCLCs. Furthermore, the relationship between EGFR activity and E-cadherin status needs to be elucidated. E-cadherin, being physically associated with EGFR [5, 11, 13], affects EGFR activity [4]. However, the role of E-cadherin as a positive [10, 11] or a negative [5, 12, 13] regulator in EGFR signaling is still controversial.

We generated the EMT cell line, which was induced by E-cadherin knockdown. In the EMT-induced cells, EGFR dependent signaling was most distinctly altered, as determined by the screening of multiple key signaling kinases (Fig. 3). Consistently, high EGFR signaling in the EMT-induced cells was associated with the EMT phenotypes, as supported by the results demonstrating that the inhibition of EGFR dependent MEK/ERK signaling either by a chemical or genetic approach abrogated the invasive phenotype (Fig. 5) and reduced the expression of the EMT-associated genes (Figs. 4 and 5). These results suggest that E-cadherin negatively regulates EGFR signaling, and its deregulation is responsible for the acquisition of metastatic properties. Consistently, the significance of MEK/ERK signaling in EMT-associated process has been studied not only in normal development but also cancer metastasis. For example, ERK activation promotes the initiation of epithelial tubule development via inducing EMT-associated morphological changes [38] and contributes to metastasis and invasion through inducing EMT [36, 37].

Our data suggest that EGFR-dependent signaling would be an important therapeutic target for NSCLC treatment, based on the results such as the requirement of MEK/ERK signaling in the invasive properties of EMT-induced A549 cells (Fig. 5) and the local activation of ERK at the invasive tumor fronts (Fig. 7). To support this notion, clinical trial of MEK inhibitors to target ERK activity such as Selumetinib [39], and PD0325901 [40] in advanced NSCLCs have been undertaken (http://clinicaltrials.gov).

Considering that circulating tumor cells (CTCs), of which number in the blood is closely associated with the patient survival [41], are presumably originated from EMT [42], inhibition or delay of forming “disseminating tumor cells at the invasive tumor fronts with dedifferentiated phenotype” [16] would be a valid strategy for lowering CTCs. Thereby, clinical application of MEK inhibitors has been attempted with PI3K inhibitors to control circulating tumor cells (CTCs) in breast cancer patients. Interestingly, in addition to the number of CTCs, treatment with both MEK and PI3K inhibitors reduces the ratio of mesenchymal type to epithelial type CTCs [43], implying that MEK inhibitor to target ERK may be more effective to mesenchymal type than epithelial type CTCs.

In summary, we show that loss of E-cadherin results in activation of MEK/ERK signaling, showing the positive correlation between loss of E-cadherin and ERK activation. In addition to A549 cell line, the positive correlation was also observed in NSCLC tissues, in which invading tumor cells were strongly stained for phospho-ERK1/2 and weakly stained for E-cadherin, indicating that the mechanical relation between E-cadherin and ERK in an A549 model cell line is relevant in NSCLC, demonstrating the importance of aberrant ERK activation in the invading tumor cells with EMT phenotypes.

## MATERIALS AND METHODS

### Plasmids and Reagents

shRNA plasmid targeting *CDH1* (TRCN 39665) and non-targeting shRNA control plasmid (SHC002) were purchased from Sigma-Aldrich. MK2206 (Cat# S1078) and PD0325901 (Cat# S1036) were purchased from Selleck Chemicals (Houston, TX, USA).

### Trans-well invasion assay

Trans-wells (6.5 mm) with 8 μm pore polycarbonate membrane inserts (Corning, NY, USA) were embedded with 120 μg matrigel (BD Biosciences, San Jose, CA, USA) and 100 μg gelatin (Sigma-Aldrich, St Louis, MO, USA) in DMEM. Cells (1x10^5^ per well) were added into the Matrigel-embedded inserts (the top chambers) in 0.5% FBS media, and the inserts were placed into the bottom chambers containing 10% FBS media. Cells were allowed to invade through the Matrigel-embedded barrier. After 24 ~ 48 hour incubation, the membranes were fixed with 4% formaldehyde in PBS for 10 min, and the invading cells on the underside of the membranes were stained with 0.1% crystal violet. The images were taken with light microscope imaging system (ProgRes C3, Jenoptik, Jena, Germany).

### Real-time PCR

Total cellular RNA was extracted using Trizol, followed by RT-PCR to generate the first strand cDNA, and the cDNA was subjected to SYBR Green-based Real-time PCR (Roche LightCycler 480 II). Primer sequences are described in Supplemental Table 1A.

### siRNA

Cells were transfected with 20 nM siRNA using Dharmafect (Thermo Scientific, Waltham, MA). Sequences of siRNA are described in Supplemental Table 1B.

### Statistical analysis

The graphical data were presented as mean ± S.E.M. Statistical significance among the three groups and between groups was determined using one-way or two-way analysis of variance (ANOVA) following Bonferroni post-test and Student's t-test respectively. Significance was assumed for p < 0.05 (*), p < 0.01 (**), p < 0.001 (***).

### Additional Materials and Methods

Methods of Immunoblotting and Immunofluorescence cytochemistry (IFC), Immunohistochemistry (IHC), Cell culture, and Zymography are available in Supplementary Materials and Methods.

## Supplemental Figures, Methods and Tables




