# Supplementary material for: Loss of E-cadherin activates EGFR-MEK/ERK signaling, which promotes invasion via the ZEB1/MMP2 axis in non-small cell lung cancer
Source: Oncotarget. 2013 Nov 29;4(12):2512–22. doi: 10.18632/oncotarget.1463 (PMC3926845; doi:10.18632/oncotarget.1463)
Supplement: Supplementary file 1 [file oncotarget-04-2512-s001.pdf]

Loss of E-cadherin activates EGFR-MEK/ERK signaling, which promotes invasion via the ZEB1/MMP2 axis in non-small cell lung cancer - Bae et al

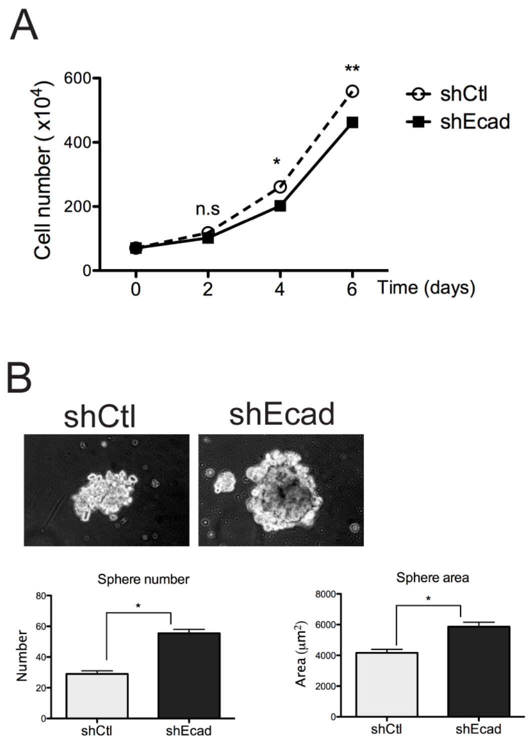

Figure S1: The EMT-associated phenotypes of shEcad cells A. Cell proliferation was determined by cell counting at 2, 4, and 6 days after plating. *Points*, means ± SE. **P* < 0.05; ***P* < 0.01. B. Cells were cultured in spheroid culture condition. Tumor sphere formation was monitored. The number and area of spheres were determined. *Columns*, means ± SE. **P* < 0.05.

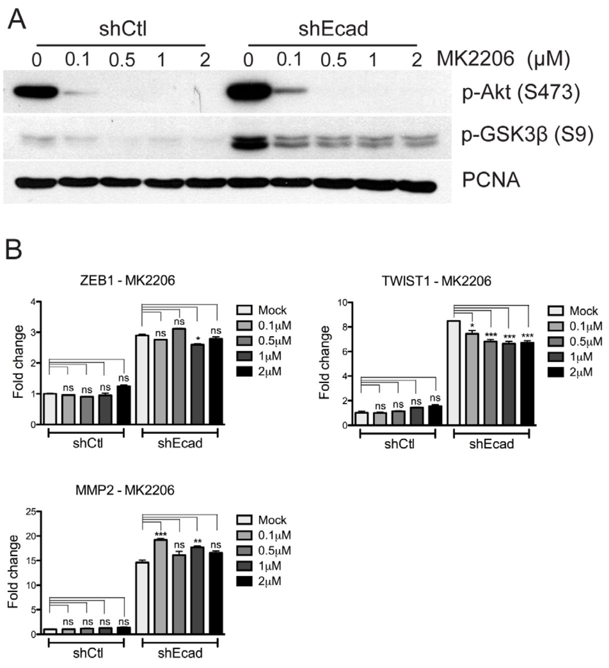

Figure S2: Akt is not responsible for elevated expression of the EMT-associated genes. A and B. Cells were treated with the indicated concentration of MK2206 for 4hr (A) or 24hr (B) and the cell lysates were harvested for protein analysis (A) or RNA analysis of *ZEB1, TWIST1* and *MMP2* (B). Inhibition of Akt by MK2206 was determined by immunolbotting against phospho-Akt and phospho-GSK3β, a substrate of Akt (A). PCNA was used as a loading control. *Columns*, means ± SE. **P* < 0.05; ***P* < 0.01; ****P* < 0.001.

SUPPLEMENTARY MATERIALS AND METHODS

Immunoblotting and Immunofluorescence cytochemistry (IFC)

Immunoblotting analysis was performed as described previously (10). Antibodies used in immunoboltting, E-cadherin (catalog# 4065), N-cadherin (catalog# 4061), phospho-EGFR (catalog# 3777), phospho-p53 (catalog# 9281), phospho-GSK3β (catalog# 9336), phospho-ERK1/2 (catalog# 9106), phospho-Akt (catalog# 4060) were purchased from Cell Signaling. α-Tubulin (sc-8035), EGFR (sc-03), β-Actin (sc-47778), PCNA (sc-56) were purchased from Santa Cruz Biotechnology. β-Catenin (catalog# 610153) was purchased from BD bioscience. For Immunofluorescence cytochemistry (IFC), cells were plated on glass coverslips and allowed to grow for overnight. For E-cadherin staining, cells were washed in PBS, fixed with 4% paraformaldehyde for 10min at room temperature, and then permeabilized with 0.1% Triton X-100 for 2min. After blocking with 3% BSA for 1hr, cells were incubated with diluted (1:200) E-cadherin antibody for 1hr. Cells were washed in PBS followed by addition of secondary antibody conjugated to a flurochrome (Jackson Laboratory) and DAPI in blocking solution for 1hr. Cells were washed in PBS and cover-slipped, and examined with a fluorescent microscope (Olympus, BX 53). Antibody used in IFC, E-cadherin (catalog# 4065) was purchased from Cell Signaling.

Immunohistochemistry (IHC)

E-cadherin (catalog# 610181) and phospho-ERK1/2 (catalog# 4370) were purchased from BD bioscience and Cell Signaling respectively. Tumor specimens and technical service of immunohistochemistry (IHC) were supported by Super Bio Chips, CO., South Korea. E-cadherin (1:200) and phospho-ERK1/2 (1:100) were stained as described in www.tissue-array.com. IHC results were reviewed by the pathologist in Samsung Medical Center, South Korea.

Cell culture

A549 cells were viral-transfected with shControl or shEcadherin vectors and subsequently selected with puromycin (3 ug/ml) to generate stable cell line, and were grown in DMEM (Gibco) supplemented with 10% FBS (Gibco) and 0.1% gentamycin. For tumor sphere culture, cells were trypsinized and suspended in serum-free DMEM/F12 media containing 2 % B27, 20ng/ml EGF, 20ng/ml bFGF, 8mM Hepes buffer and 0.1% gentamycin, and then cultured in low attachment culture dish (50, 000 cells / 35 mm dish).

Zymography

Conditioned media by cells were concentrated using centricon (Millipore, 30kDa cut), and added with non-reducing sample buffer (without DTT). The samples were loaded into the SDS-PAGE gel containing gelatin B (Sigm-Aldrich). After electrophoresis, gel was washed with 2.5% Triton X-100 in Tris-HCL buffer (pH 7.5). Then the gel was incubated with reaction buffer (15 mM Nacl, 10 mM $CaCl2$ in Tris-HCl butter (pH 7.5)) overnight at room temperature to induce gelatin lysis by MMP2 or MMP9, and then stained with Coomassie brilliant blue for 30 min followed by destaining.
