# Supplementary material for: Loss of E-cadherin activates EGFR-MEK/ERK signaling, which promotes invasion via the ZEB1/MMP2 axis in non-small cell lung cancer
Source: Oncotarget. 2013 Nov 29;4(12):2512–22. doi: 10.18632/oncotarget.1463 (PMC3926845; doi:10.18632/oncotarget.1463)
Supplement: Supplementary file 2 [file oncotarget-04-2512-s002.pdf]

Loss of E-cadherin activates EGFR-MEK/ERK signaling, which promotes invasion via the ZEB1/MMP2 axis in non-small cell lung cancer - Bae et al

Supplementary Table 1: Oligonucleotides used in this study

A. Primer sequences

Genes	Primer sequences (5` -> 3`)
ACTA2	Forward: acctacaacagcatcatgaagtg
	Reverse: cttcatcgtattcctgtttgctg
ACTB	F: gtcctctcccaagtccacac
	R: gggagaccaaaagccttcat
CDH1	F: tgcccagaaaatgaaaaagg
	R: gtgtatgtggcaatgcgttc
CDH2	F: gacaatgcccctcaagtgtt
	R: ccattaagccgagtgatggt
ERK1	F: gacctgctggaccggatgttaacc
	R: ctcctggaagatgagctccttcag
ERK2	F: gaacaggctgttcccaaatgctgac
	R: ccatgtcgaacttgaatggtgcttc
MMP2	F: ctcccggaaaagattgatg
	R: ggtgctggctgagtagat
MMP9	F: gtgagttgaaccaggtggaccaag
	R: cactcctccctttcctccagaac
SLUG	F: ctttttcttgccctcactgc
	R: gcttcggagtgaagaaatgc
TWIST1	F: gtcttacgaggagctgcagac
	R: tgagccacatagctgcagcttg
VIMENTIN	F: tgagattgccacctacaggaagc
	R: cgtgatgctgagaagtttcgttg
ZEB1	F: gtagaggatcagaatgactctg
	R: ccagaatgtaatcgcatgtgttc

B. siRNA sequences

Genes	siRNA sequences (5` -> 3`)
ZEB1	acacaagcgagaggaucau
TWIST1	cugaacguuguuuguguu
ERK1	cucucuaaccggcccaucu
ERK2	cagaucuuuacaagcucuu
MMP2	cugcaaacaggacauugua
